# Middle East respiratory syndrome coronavirus infection in non-camelid domestic mammals

**DOI:** 10.1080/22221751.2018.1560235

**Published:** 2019-01-16

**Authors:** Ahmed Kandeil, Mokhtar Gomaa, Mahmoud Shehata, Ahmed El-Taweel, Ahmed E. Kayed, Awatef Abiadh, Jamel Jrijer, Yassmin Moatasim, Omnia Kutkat, Ola Bagato, Sara Mahmoud, Ahmed Mostafa, Rabeh El-Shesheny, Ranawaka APM Perera, Ronald LW Ko, Nagla Hassan, Basma Elsokary, Lotfi Allal, Ahmed Saad, Heba Sobhy, Pamela P. McKenzie, Richard J. Webby, Malik Peiris, Mohamed A. Ali, Ghazi Kayali

**Affiliations:** a Center of Scientific Excellence for Influenza Virus, National Research Centre, Giza, Egypt; b Nature Link, Sfax, Tunisia; c Institute of Medical Virology, Justus Liebig University Giessen, Giessen, Germany; d St. Jude Children’s Research Hospital, Memphis, TN, USA; e School of Public Health, University of Hong Kong, Sandy Bay, Hong Kong; f General Organizations of Veterinary Services, Ministry of Agriculture and Land Reclamation, Giza, Egypt; g Food and Agriculture Organization of the United Nations, Emergency Center for Transboundary Animal Diseases, Giza, Egypt; h Human Link, Baabda, Lebanon; i University of Texas Health Sciences Center, Houston, TX, USA

**Keywords:** MERS-CoV, surveillance, serology, Egypt, Tunisia, Senegal, sheep

## Abstract

Dromedary camels are natural host of the Middle East respiratory syndrome coronavirus (MERS-CoV). However, there are limited studies of MERS-CoV infection of other domestic mammals exposed to infected dromedaries. We expanded our surveillance among camels in Egypt, Tunisia, and Senegal to include other domestic mammalian species in contact with infected camels. A total of 820 sera and 823 nasal swabs from cattle, sheep, goats, donkeys, buffaloes, mules, and horses were collected. Swabs were tested using RT-PCR and virus RNA-positive samples were genetically sequenced and phylogenetically analysed. Sera were screened using virus microneutralization tests and positive sera (where available) were confirmed using plaque reduction neutralization tests (PRNT). We detected 90% PRNT confirmed MERS-CoV antibody in 35 (55.6%) of 63 sera from sheep collected from Senegal, two sheep (1.8%) of 114 in Tunisia and a goat (0.9%) of 107 in Egypt, with titres ranging from 1:80 to ≥1:320. We detected MERS-CoV RNA in swabs from three sheep (1.2%) of 254 and five goats (4.1%) of 121 from Egypt and Senegal, as well as one cow (1.9%) of 53 and three donkeys (7.1%) of 42 from Egypt. Partial sequences of the RT-PCR amplicons confirmed specificity of the results. This study showed that domestic livestock in contact with MERS-CoV infected camels may be at risk of infection. We recommend expanding current MERS-CoV surveillance in animals to include other livestock in close contact with dromedary camels. The segregation of camels from other livestock in farms and live animal markets may need to be considered.

## Introduction

The Middle East Respiratory Syndrome (MERS) is caused by a beta-coronavirus (CoV) first detected in a Saudi male in 2012 [1]. Since then, the World Health Organization (WHO) has recorded 2260 laboratory-confirmed cases, at least 803 of these being fatal [2]. Surveys of camels in the Middle East and Africa showed that they are a natural host of MERS-CoV [2,3] and they are a source of human infection [4].

Host specificity of MERS-CoV is determined by the presence of the dipeptidyl peptidase-4 (DPP4) receptors expressed on cell surfaces. DPP4 was found to be a functional receptor for the receptor-binding S1 domain (RBD) of the MERS-CoV spike protein [5,6]. There is limited data on the susceptibility of other livestock in close contact with camels to MERS-CoV infection. A survey of goats, camels, and sheep from Jordan showed no evidence of infection [7]. Surveys on horses in the United Arab Emirates, Saudi Arabia, and Oman did not provide conclusive evidence of infection [8,9].

We expanded our MERS-CoV surveillance programme in Egypt, Tunisia, and Senegal to include other domestic livestock in contact with camels.

## Results

We carried out 17 sampling visits to mixed farms or herds where camels and other livestock were raised in Senegal and Egypt and a livestock market in Tunisia. Camels in contact with these livestock were sampled during these visits and MERS-CoV RNA was detected in camels in 13 of the 17 sampling trips while seropositive camels were detected in all trips (data not shown).

In total, 820 serum samples of livestock other than camels in contact with camels were tested for MERS-CoV antibodies by the screening microneutralization (VMN) test and 56 of them had detectable antibody to MERS-CoV. All but 2 cattle sera were from adult animals. Out of these sera, 49 were available for confirmatory testing by PRNT tests using the stringent 90% reduction of plaque numbers (PRNT90) as the criterion for evidence of neutralizing antibody. There was PRNT90 antibody to MERS-CoV in 38 adult animal sera; in 35 (55.6%) of 63 sheep sampled in Senegal, one of 107 goats sampled in Egypt and two of 114 sheep sampled in Tunisia (Table 1). The PRNT90 antibody titres of these positive sera ranged from 1:80 to ≥1:360. Sera positive for MERS-CoV antibodies (37 sheep sera from Senegal, one sheep from Tunisia and one goat from Egypt) with antibody titres ranging from 1:20 to >1:320 were selected for testing by microneutralization tests for bovine coronavirus (BCoV). Ten of the MERS-CoV antibody positive sheep sera from Senegal and the sheep serum from Tunisia had no detectable antibody to bovine coronavirus. Even those sera with antibody to bovine coronavirus had antibody titre that were lower than that observed for MERS-CoV (Table S1).

**Table 1.** RNA detection in nasal swabs and sera tested for MERS-CoV antibodies by microneutralization (VMN) and confirmed by 90% plaque reduction neutralization (PRNT90) in samples obtained from domestic mammals in contact with camels in Egypt and Tunisia.

Species	Number of nasal swabs	MERS CoV positive	Serum samples tested by VMN	VMN positive	Serum samples Tested by PRNT	PRNT positive
*Egypt*						
Sheep	191	2	195	1	0	–
Goats	112	4	107	3	3	1
Cattle	53	1	52	4	1	0
Horse	6	0	6	0	–	–
Donkey	42	3	41	3	3	0
Buffalo	5	0	5	0	–	–
*Tunisia*						
Sheep	114	0	114	3	3	2
Goats	204	0	204	5	3	0
Cattle	15	0	15	0	–	–
Horse	2	0	2	1	0	–
Donkey	2	0	2	0	–	–
Mule	5	0	5	0	–	–
*Senegal*						
Sheep	63	1	63	35	35	35
Goats	9	1	9	1	1	0

MERS-CoV RNA was detected in 12 of 823 swabs collected from sheep, goats, cattle, buffalo, horses, donkeys and mules (Table 1), virus RNA being detected in three sheep, five goats, three donkeys and one cow from Egypt or Senegal. All animals were adults except for 1 juvenile goat and another juvenile donkey. All swabs that tested positive by upE rtRTPCR had CT values ≥38 indicating low virus load and were confirmed by at least one additional RT-PCR assay targeting the ORF1a, N or S genes (Table 2). Four of these upE rtRT-PCR positive samples were ORF1a rtRTPCR positive while the others were confirmed by N or S (RBD) gene RT-PCR assays. Five samples were also confirmed by sequencing the RBD region of the spike gene. The host species of each MERS-CoV positive sample were confirmed by DNA barcoding to exclude possibility of mix up of camel swabs with those of other domestic livestock. Those RT-PCR positive animals were MERS-CoV seronegative. None of the swabs collected from sheep or goats in Tunisia was rtRT-PCR positive.

**Table 2.** Detection of MERS CoV RNA in nasal swabs in Egypt.

Sample	upE	Orf1a	Nseq	S (RBD)
MERS CoV/donkey/Egypt/2213/2015	Pos	Neg	Neg	Pos
MERS CoV/donkey/Egypt/3933/2016	Pos	Neg	Neg	Pos
MERS CoV/donkey/Egypt/3976/2016	Pos	Pos	ND	ND
MERS CoV/cattle/Egypt/2257/2015	Pos	Neg	Neg	Pos
MERS CoV/sheep/Egypt/3838/2016	Pos	Pos	Pos	ND
MERS CoV/sheep/Egypt/3853/2016	Pos	Pos	Pos	ND
MERS CoV/goat/Egypt/2284/2015	Pos	Neg	Neg	Pos
MERS CoV/goat/Egypt/3850/2016	Pos	Neg	Neg	Pos
MERS CoV/goat/Egypt/3893/2016	Pos	Neg	Neg	Pos
MERS CoV/goat/Egypt/3975/2016	Pos	Pos	ND	ND
MERS CoV/sheep/Senegal/M46/2017	Pos	Neg	Pos	Neg
MERS CoV/goat/Senegal/M178/2017	Pos	Pos	Neg	Pos

Note: upE positive samples were confirmed by at least another assay targeting the Orf1a, N, or S (RBD) genes. ND: Not done.

In order to provide additional confirmation of the specificity of the rtRT-PCR positive results for MERS-CoV RNA, the region of the RT-PCR amplified spike RBD domain was sequenced. Sequence alignment of positive Egyptian samples from goats, cattle, and donkeys collected in 2015 or 2016 against the first human MERS isolate (EMC/2012) which was used as the positive control in RT-PCR revealed a point mutation A431 T in the S(RBD) gene (Figure S1). This sequence heterogeneity reduces the possibility of PCR contamination as an explanation for RT-PCR positivity.

Alignment of the DPP4 sequences of various domestic mammalian species revealed that DPP4 residues interacting with the MERS CoV spike RBD were identical in camel, sheep, and goat (Figure S2). Equine DPP4 had two amino acid mutations at positions 288 (V288T) and 289 (P289A).

## Discussion

MERS-CoV is a zoonotic virus and dromedary camels are a source of human infection [10]. While there have been some surveillances of other domestic livestock species during the initial search for the natural host of MERS-CoV in 2013-14, there is a paucity of a systematic investigation of livestock in close proximity to infected dromedaries. We have previously reported a seropositive sheep raised in contact with camels in Egypt [11]. However, surveillance in mammals in an area where MERS-CoV infected camels in Jordan yielded negative results [7]. MERS-CoV infection was not detected in equids from the United Arab Emirates where camel and human infection were previously reported [9]. Although a study of horses in Saudi Arabia found no convincing evidence of MERS-CoV infection in these horses, one of 243 equine sera had a PRNT90 titre of 1:40 [8].

In this paper, we provide the first convincing evidence of naturally acquired MERS-CoV infection in sheep raised in prolonged close proximity with dromedary camels in Senegal with a PRNT90 seroprevalence of 55.6% with PRNT90 antibody titres ranging from 1:80 to ≥1:320. Evidence of PRNT90 antibody was also found in 2 of 114 sheep sera collected from Tunisia and 1 of 112 goats in Egypt. Since not all VMN-positive sera from Egypt had sufficient serum to be tested in the confirmatory PRNT90 assay, we may have under-estimated the true sero-prevalence in livestock sampled in Egypt. The serological testing for MERS-CoV was carried out using a virus isolated in Egypt (camel/Egypt/163/14) (for the MN test) and prototype strain EMC (for the PRNT test). We have previously shown that MERS-CoV from East, West, and North Africa are antigenically indistinguishable from those in the Arabian Peninsula and prototype strain EMC [12].

We have previously shown that antisera to closely related betacoronaviruses such as bovine coronavirus, mouse hepatitis virus, or SARS coronavirus do not cross-react with MERS-CoV in neutralization tests [13]. Nevertheless, because BCoV is a betacoronavirus known to be endemic in ruminants including sheep, we tested for VMN antibody to BCoV in selected MERS-CoV antibody positive sheep sera [14]. Our data suggest that cross-reactive BCoV antibody does not explain the observed antibody titres to MERS-CoV. The possibility of a hitherto undiscovered coronavirus cross-reacting with MERS-CoV cannot however be excluded. Our findings may be evidence of repeated cross-species transfer of MERS-CoV from dromedaries to sheep rather than natural self-sustained transmission between sheep.

We detected MERS-CoV RNA by rtRT-PCR in sheep, goats, and donkeys in farms in Egypt and in a sheep and goat in nomadic herds in Senegal, all of these animals being in close contact with dromedaries. These animals were sero-negative, as expected, as serological responses only appear 2–3 weeks after infection by which time virus RNA will usually be undetectable. The rtRT-PCR positive animals were asymptomatic as is the case for camels infected with MERS-CoV. The identity of the host species of the swabs positive for virus RNA was additionally confirmed by DNA barcoding in order to assure that no mix up with camel swabs had occurred during sampling or sample handling. Finding MERS-CoV RNA in upper respiratory tract swabs may simply represent exposure and environmental contamination from infected camels or environments contaminated with camel excretions rather than convincing evidence of active infection. In contrast, evidence of MERS-CoV neutralizing antibody implies evidence of active infection by MERS-CoV or a closely related virus. Attempts to culture the viruses detected in the study or to obtain full-genome sequences were not successful hence limiting our ability to further verify the findings of this study. It is important to note that this evidence comes from countries where human infection with MERS-CoV was never reported locally and that evidence of MERS-CoV infection in non-camelid mammals was never reported in countries where human infections are common.

MERS-CoV was detected in species that express DPP4 receptor essential for MERS-CoV attachment and internalization into mammalian cells [6] and the DPP4 sequences of those species were similar to that found in camels, especially in regions where interaction with the MERS-CooV spike protein RBD occurs. Two mutations were detected in the horse DPP4 sequences.

Interestingly, all sequences of MERS-CoV obtained from the domestic livestock in contact with camels in Egypt possessed mutation A431T in the S gene (RBD) that was also detected in a sequence from a contact camel. Experimental inoculation of sheep and horses with the first prototype isolate of MERS-CoV (HCoV-EMC/2012) showed no infection despite that the same virus was able to infect pigs and llamas [15,16]. This may be due to the absence of the A431T mutation in the virus used in those studies. This mutation may be a key to enabling MERS-CoV infection in non-camelid domestic species and this requires further study. Future experimental infections of livestock need to be conducted also using MERS-CoV of African origin.

In summary, we provide evidence of MERS-CoV, or a very closely related virus, infection of domestic livestock (other than camels) in close contact with camels, suggesting that spill over infection to other livestock may occur. Our data does not prove MERS-CoV can be sustained by transmission within these other livestock species in the absence of camels as the primary source of infection. However, if other domestic livestock such as sheep may be infected by MERS-CoV, they may also be a risk factor for human infection. Our findings highlight the need for further field studies of domestic livestock, especially those that are in close contact with dromedary camels and also the need for experimental studies infecting livestock such as sheep using MERS-CoV isolates from Africa. Our findings may raise the need to segregate other livestock from dromedary camels in farms and live animal markets.

## Materials and methods

### Samples, detection, and sequencing

In Egypt, 406 sera and 409 nasal swabs were collected from 315 adult (>1 year) and 92 juvenile cattle, sheep, goats, donkeys, buffaloes, and horses in close contact with camels from farms in three governorates: Sharkia (Nile Delta region), Beheira (Northern Egypt), and Luxor (Southern Egypt) monthly between August 2015 and September 2017. In Tunisia, 342 sera and 346 nasal swabs were obtained from 321 adult and 25 juvenile cattle, sheep, goats, donkeys, mules, and horses in contact with camels at a major livestock market in Southern Tunisia during December and January of 2016 and 2017. In Senegal, 72 sera and 72 nasal swabs were collected from 61 adult and 11 juvenile sheep and goats in contact with pastoral camels in the Northwest of the country during August 2017.

Nasal swabs were screened by real-time reverse transcription PCR (rtRT-PCR) targeting upstream of E gene of MERS-CoV [17]. Positive samples were confirmed using first the Open Reading Frame (ORF) 1a. When ORF1a testing was negative, the N gene-based PCR assay was then performed as recommended by the WHO [18]. Samples not confirmed by ORF1a or N were tested by an in-house RT-PCR for the spike protein receptor binding domain (RBD) (see below) and PCR products to confirm specificity of the RT-PCR assay.

A partial fragment in spike RBD was amplified using pre-RBD_MERS_F (GAA TCT GGA GTT TAT TCA GTT TCG T) and pre-RBD_MERS_R (ACG GCC CGA AAC ACC ATA G) primers in the first round using one step RT-PCR kit (QIAGEN, Germany). The PCR products were then subjected to a second PCR using RBD_MERS_F: (CGA AGC AAA ACC TTC TGG CT) and RBD_MERS_R: (ATA TTC CAC GCA ATT GCC TA) using Phusion High Fidelity PCR Master Mix Kit (Thermo Scientific, USA). The phylogenetic tree was constructed using MEGA6 programme by applying the neighbor-joining method with Kimura’s two-parameter distance model and 1000 bootstrap replicates [19]. Sequence alignment was performed using BioEdit 7.0 (ref. [20]). DPP4 amino acid reference sequences of camels, sheep, goats, cattle, horses, and donkeys were downloaded from GenBank and aligned using BioEdit.

### Serology tests

*Microneutralization:* The methods used have been described before [21]. MERS-CoV (strains: EMC and camel/Egypt/163/14) and bovine coronavirus (BCoV) (ATCC BRCV-OK-0514-2) were used. Vero cells (ATCC CCL-81) were used for MERS-CoV and HRT-18G cells (obtained from ATCC) for BCoV. Serum dilutions were mixed with equal volumes of 200 tissue culture infective dose 50 of virus and incubated for one hour at 37°C. The virus-serum mixture was then added in quadruplicate to cell monolayers in 96-well microtitre plates. After one hour of adsorption, the virus-serum mixture was removed and 150 μl of fresh culture medium was added to each well and the plates incubated at 37°C in 5% CO_2_ in a humidified incubator. A virus back-titration was performed without immune serum to assess input virus dose. Cytopathic effect (CPE) was read at three days post-infection for MERS-CoV and four days post-infection for BCoV. The highest serum dilution that completely protected the cells from CPE in half of the wells was defined as the neutralizing antibody.

*Plaque reduction neutralization (PRNT)*: The PRNT assays were performed on heat inactivated sera in 24-well tissue culture plates in duplicate for each serum dilution as previously described [21]. Briefly, two-fold serum dilutions were incubated with 40–60 plaque-forming units of MERS-CoV (strain EMC) virus for 1 h at 37°C. Then the virus and serum mixture were added to a pre-formed Vero cell monolayer and incubated for 1 hr at 37°C in a 5% CO_2_ incubator. Then, the supernatant was removed and the cells were overlaid with 1% agarose (SeaKem LE Agarose, Lonza, Switzerland) in cell culture medium (Minimum Essential Medium with 2% foetal bovine serum). After three days incubation the plates were fixed and stained. Endpoint PRNT antibody titres were defined as the highest serum dilutions that resulted at ≥50% (PRNT50) and ≥90% (PRNT90) inhibition of the number of plaques, respectively. Only the PRNT90 data is shown because this is the more stringent end point for virus neutralization.

### RBD sequencing

A partial fragment in the spike protein RBD was amplified with pre-RBD_mers_F (GAA TCT GGA GTT TAT TCA GTT TCG T) and pre-RBD_mers_R (ACG GCC CGA AAC ACC ATA G) primers using a One-step RT-PCR kit (QIAGEN, Hilden Germany), in 25 µl reactions [5 µl of 5X reaction buffer, 1 µl dNTPs, (10 mM) 1 µl enzyme mix, 1.5 µl (10 µM) forward primer, 1.5 µl (10 µM) reverse primer, 10 µl ddH2O, and 5 µl of sample RNA]. The PCR cycler conditions were 50°C for 30 min, 95°C for 15 min, then 45 cycles of 94°C for 15 s, 50°C for 30 s and 72°C for 90 s, and finally 72°C for 10 min. The PCR product was then subjected to a second PCR using RBD_mers_F (CGA AGC AAA ACC TTC TGG CT) and RBD_mers_R (ATA TTC CAC GCA ATT GCC TA) primers and Phusion High Fidelity PCR Master Mix Kit, (Thermo Scientific, Waltham, MA, USA). A 25 µl reaction included 12.5 µl of 2X phusion master mix, 1.5 µl (10 µM) forward primer, 1.5 µl (10 µM) reverse primer, 6.5 µl H_2_O, and 3 µl of PCR product of the first round. The PCR cycler conditions were 98°C for 30 s, then 40 cycles (98°C for 10 s, 48°C for 30 s, 72°C for 60 s), then 72°C for 10 min. The final PCR product was gel purified then, sequenced with the same primers at the Macrogen sequencing facility (Macrogen, South Korea).

DPP4 amino acid sequences of camels, sheep, goat, cattle, horse, and donkey (accession numbers AIG55259, AIG55264, AIG55261, NP776464, XP005601601, and XP014715582, respectively) were downloaded from GenBank and aligned using BioEdit.

### Host DNA barcoding

Cytochrome oxidase subunit 1 barcoding was used to confirm species from which MERS CoV positive swabs were obtained as previously described [22]. The amplifed PCR products were sequenced and sequences were compared with available sequences in GenBank. Ethical approval was received from the St. Jude Children’s Research Hospital IACUC.

### Data availability

The nucleotide sequences obtained in this study are available from GenBank under accession numbers MF318497, MF318498, MF318499, MF318500, MF318501, MF318502, MF318503, and MF318504.
